# Long-term outcomes after cytoreductive nephrectomy and thrombectomy of patients with metastatic renal cell carcinoma with venous tumor thrombus: a retrospective study from a large Chinese center

**DOI:** 10.1186/s12957-023-03048-z

**Published:** 2023-06-07

**Authors:** Kewei Chen, Zhuo Liu, Yuxuan Li, Xun Zhao, Yu Zhang, Hai Bi, Guoliang Wang, Cheng Liu, Xiaojun Tian, Hongxian Zhang, Lulin Ma, Shudong Zhang

**Affiliations:** Department of Urology, Peking University Third Hospital, Haidian District, Beijing, 100191 People’s Republic of China

**Keywords:** Metastatic renal cell carcinoma, Tumor thrombus, Cytoreductive nephrectomy, Overall survival

## Abstract

**Background:**

Targeted therapy combined with immunotherapy is the current first-line treatment for metastatic renal cell carcinoma (mRCC), but patients with tumor thrombus (TT) may suffer from lower limb edema or even sudden cardiac death, so the purpose of this study is to investigate the efficacy and safety of surgical treatment in patients with mRCC and TT and explore worse factors to affect the prognosis in this series of patients.

**Patients and methods:**

A total of 85 mRCC patients with TT who received cytoreductive nephrectomy and thrombectomy at our medical center from 2014 to 2023 are included. All patients received postoperative systemic therapy. Overall survival (OS) is defined as the time from surgery to death due to any reason or the last follow-up. Kaplan–Meier analysis was performed to evaluate OS and differences among groups were tested by log-rank. Multivariable Cox proportional hazards analysis was performed to ascertain independent relationships between clinicopathological factors and OS.

**Results:**

The median age of patients was 58 years old. Eleven patients (12.9%) had no symptoms, 39 patients (45.9%) had local symptoms, 15 patients (17.6%) had systemic symptoms, and 20 patients (23.5%) had both. Mayo grade of TT was 0, 1, 2, 3, and 4 for 12, 27, 31, 7, and 8 patients respectively. Fifty-five patients had lung metastasis, 23 had bone metastasis, 16 had liver metastasis, 13 had adrenal metastasis, and 9 had lymph node metastasis. Of all patients, 17 patients had multiple metastases. The median operation time is 289 min and the median intraoperative hemorrhage is 800 ml. Twenty-eight patients experienced postoperative complications, 8 of which were serious complications of modified Clavien grade III or higher. The median OS of all patients was 33 months and median follow up time was 26 months. In multivariate analysis, systemic symptom (*p* = 0.00753), pathological type (*p* = 0.0166), sarcomatous degeneration (*p* = 0.0334), and perirenal fat infiltration (*p* = 0.0202) are independent predictors of OS.

**Conclusion:**

Cytoreductive nephrectomy and thrombectomy is relatively safe and effective for patients with mRCC accompanied by TT. In this series of patients, the worse prognosis is associated with systemic symptoms, non-clear cell carcinoma, sarcomatous degeneration and perirenal fat infiltration.

## Introduction

Inferior vena cava (IVC) invasion is one of the characteristics of renal cell carcinoma (RCC), accounting for approximately 4–10% patients [1, 2]. About 30% of patients with tumor thrombus (TT) have distant metastasis and the treatment of such patients is still controversial [3]. In the past decade, benefiting to the in-depth study of the molecular mechanism of RCC, Sunitinib, and Temsirolimus have emerged as a front-line standard of mRCC patients, and Bevacizumab or interferon is likely to be the next approved first-line treatment [4, 5]. Two randomized clinical studies, SURTIME [6] and CARMENA [1] showed that cytoreductive nephrectomy was of limited value in the treatment of mRCC. Therefore, surgical treatment seems to be given unduly high status. However, it can cause pulmonary embolism due to the shedding of brittle tissue of TT, even causes sudden cardiac death because of the rapid growth of TT [7]. In addition, patients with IVC TT are prone to vena cava obstruction symptoms, such as lower limb edema, ascites, impaired renal function, and Bucha’s syndrome, which diminished the quality of life. It can effectively remove TT, increase the returned blood volume and reduce the potential risk of pulmonary embolism by cytoreductive nephrectomy and thrombectomy. It has significant clinical implications and has the effect of alleviating patients’ symptoms. However, the safety and effectiveness of cytoreductive nephrectomy for mRCC patients with IVC TT need to be clarified. At present, there are few studies on sequential targeted therapy or both immunotherapy and targeted therapy after cytoreductive nephrectomy for mRCC patients with TT [8–10], and the result of long-term follow-up is not clear. We conducted a retrospective analysis of long-term outcomes of patients underwent cytoreductive nephrectomy and thrombectomy followed systematic treatment to verify feasibility and safety of surgical treatment and explore worse factors to affect the prognosis in this series of patients.

## Patients and methods

From 2014 to 2023, 98 RCC patients with venous TT received a diagnosis of radiographically distant metastases at our medical center. Three patients did not get surgical or systemic therapy due to the rapid advancement of their mRCC, and 4 patients received simple systemic therapy, including targeted therapy and immunotherapy. Six patients undergoing neoadjuvant treatment were excluded. All 85 patients included in our research received cytoreductive nephrectomy with thrombectomy and received postoperative systemic therapy including targeted therapy or both targeted therapy and immunotherapy. Clinicopathological data for the included individuals were available and were obtained from their medical records.

All patients underwent routine lab tests, abdominal computed tomography (CT) or magnetic resonance imaging (MRI) before surgery to assess the status of the primary renal tumor and venous TT. In these 85 patients, the general extension of TT to IVC was assessed by enhanced CT or MRI on the sagittal plane and was confirmed during surgery. To identify distant metastases, all patients had bone scan, positron emission tomography computed tomography (PETCT), brain and chest CT, or MRI. The American Society of Anesthesiologists (ASA) grading is used to assess the patient's overall preoperative status [11]. According to the WHO 2016 version, the pathological and nuclear grades of the renal tumor are categorized [12].

The Mayo grading system is used to classify the TT [13]. In patients with a range of Mayo grade TT, several surgical techniques were established, and the surgical indications were based on the patient’s strong performance, comparatively small number of extrarenal illnesses, and low risk of postoperative complications. According to the modified Clavien system [14], perioperative complications that occurred within 30 days of cytoreductive nephrectomy with thrombectomy are categorized. The course of treatment for Mayo grade 0 TT is same to cytoreductive nephrectomy. In grade I TT, the affected portion of the IVC wall was removed and reconstructed after the IVC was blocked to remove the thrombus. For grade II TT, the IVC was cut to remove the thrombus after the distal vena cava of the TT, the opposing renal vein, and the proximal IVC were each occluded in turn. For grade III TT exceeded the hepatic vein, the liver ligament was severed in order to separate the liver from the diaphragm upstream. After the Pringle Manouver technique is isolated and the first hepatic portal is blocked, the IVC was released and blocked to remove the TT [15]. For grade IV TT not entering the right atrium, the central tendon of the diaphragm was cut around the vena cava or the diaphragm was directly cut and then TT was gently pushed into the IVC to change it to the lower diaphragm in order to further remove the TT. As systemic treatment for mRCC after surgery, immunotherapy drugs or targeted drugs are provided to selected patients. Targeted drugs include Sunitinib, Sorafenib, Axitinib, Everolimus, and Pezopanib. Immunotherapy includes Pembrolizumab, Tislelizumab, Toripalimab, and Durvalumab. Experienced chemotherapists provide drug use guidance to patients with mRCC and adjust the dosage of drugs according to the adverse events. The patients have laboratory examination and radiographical examination per 8–12 weeks after surgery.

Overall survival (OS) is defined as the time from cytoreductive nephrectomy and thrombectomy to death due to any reason or the last follow-up. OS from the date of surgery until death based on TT level or risk grouping was estimated by Kaplan–Meier analysis and differences among groups were tested by log-rank. Multivariable Cox proportional hazards analysis was performed to ascertain independent relationships between preoperative factors and OS. Continuous variables were expressed as Mean ± SD and categorical variables were expressed as percentages. *P* < 0.05 was considered statistically significant. All data were analyzed by SPSS 22.0 software (IBM Corp, Armonk, NY, USA).

## Results

**Fig. 1:**
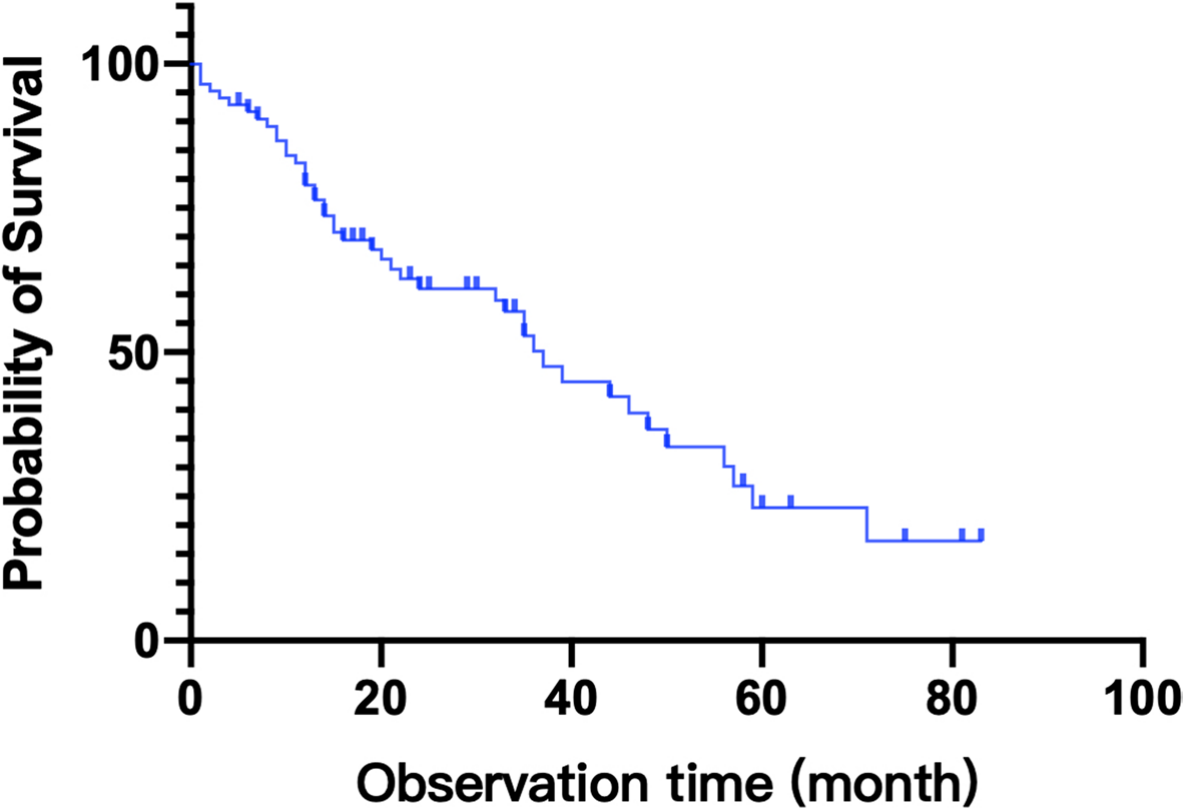
Overall survival of 85 patients with metastatic renal cell carcinoma with venous tumor thrombus who received cytoreductive nephrectomy

A total of 85 patients identified with mRCC and TT who received cytoreductive nephrectomy and thrombectomy were included. Table 1 shows the clinicopathological data of 85 patients. The median age of patients was 58 years old and the sex ratio was about 3 to 1. Of 55 patients with single metastasis, 1 patient received excision of bone metastases and 1 patient received pulmonary lobectomy. All patients with multiple metastasis did not receive surgical removal of distant metastatic tumors. All patients received post-operative systemic therapy, 67 received targeted therapy and 18 received both targeted therapy and immunotherapy. Twenty-three patients were treated with Sunitinib, 16 with Sorafenib, 25 with Axitinib, 19 with Everolimus, and 15 patients with Pezopanib. And 18 patients received immunotherapy at the same time, including 2 with Pembrolizumab, 5 with Tislelizumab, 4 with Toripalimab, and 9 with Durvalumab. Twenty-eight patients (32.9%) experienced postoperative complications. Among them, 2 cases were classified as modified Clavien grade I for wound infection. Eighteen cases were classified as modified Clavien grade II, including 6 cases treated with blood transfusion for anaemia, 3 cases of celiac fistula, 5 cases of postoperative pulmonary infection, 2 cases of postoperative lower limb venous thrombus, 1 case of atrial fibrillation and 1 case of epididymitis. Five cases were classified as modified Clavien grade III, 3 cases of pneumothorax and 2 cases of urinary fistula. Three cases were classified as modified Clavien grade IV, including 1 case of postoperative acute cerebral infarction and 2 cases of postoperative renal insufficiency. No patients were found with pulmonary embolism or sudden cardiac death during the perioperative period. The median OS of all patients was 33 months and median follow up time was 26 months. The OS of all patients was shown in Fig. 1.

**Table 1:** Clinicopathological characteristics of 85 patients with metastatic renal cell carcinoma and tumor thrombus who received cytoreductive nephrectomy and thrombectomy

Age (years, range)	58 (20–78)
Gender	
Male	65 (76.5%)
Female	20 (23.5%)
Weight (kg, range)	63 (37–112)
Height (cm, range)	167 (149–183)
Body mass index (kg/m^2^, range)	22.8 (15.2–35.0)
Symptom	
No	11 (12.9%)
Local symptom	39 (45.9%)
Systemic symptom	15 (17.6%)
Both	20 (23.5%)
Mayo grade	
0	12 (14.1%)
1	27 (31.8%)
2	31 (36.5%)
3	7 (8.2%)
4	8 (9.4%)
Side	
Left	33 (38.8%)
Right	52 (61.2%)
Tumor diameter (cm, range)	9.4 (2.8–21.1)
Major metastatic organs	
Lung	55
Bone	23
Liver	16
Adrenal	13
Lymph node	9
Single metastatic or not	
Single site	55 (64.7%)
Multiple sites	30(35.3%)
ASA classification	
1	8 (9.4%)
2	60 (70.6%)
3	15 (17.6%)
4	2 (2.4%)
Operation approach	
Open	42 (49.4%)
Laparoscopy	35 (41.2%)
Robot-assisted laparoscopy	8 (9.4%)
Ipsilateral adrenalectomy	
Yes	52 (61.2%)
No	33 (38.8%)
Partial resection of IVC	
Yes	14 (16.5%)
No	71 (83.5%)
Lymph node dissection	
Yes	43 (50.6%)
No	42 (49.4%)
Pathological type	
Clear cell carcinoma	61 (71.8%)
Papillary cell carcinoma	8 (9.4%)
Other	16 (18.8%)
Nuclear grading	
1	6 (7.1%)
2	16 (18.8%)
3	41 (48.2%)
4	22 (25.9%)
Sarcomatous degeneration	
Yes	16 (18.8%)
No	69 (81.2%)
Perirenal fat infiltration	
Yes	34 (40.0%)
No	51 (60.0%)
Preoperative Scr (ml/min/1.73 m^2^, range)	91 (32–258)
Postoperative Scr (ml/min/1.73 m^2^, range)	95 (41–480)
Operation time (min, range)	289 (135–635)
Intraoperative hemorrhage (ml, range)	800 (10–6000)
Complications	
0	57 (67.1%)
1	2 (2.4%)
2	18 (21.2%)
3	5 (5.9%)
4	3 (3.5%)
Serious complications	
Yes	8 (28.6%)
No	20 (71.4%)
Postoperative systemic therapy	
Targeted therapy	67 (78.8%)
Targeted therapy and immunotherapy	18 (21.2%)
IMDC score	
0	42 (49.4%)
1	29 (34.1%)
2	14 (16.5%)

Local symptoms include lumbago, hematuria or abdominal mass and systemic symptoms include fever, edema, or emaciation. *ASA* American Society of Anesthesiologists. *IVC* inferior vena cava. *Scr* serum creatine. Complications were classified as the modified Clavien system

**Fig. 2:**
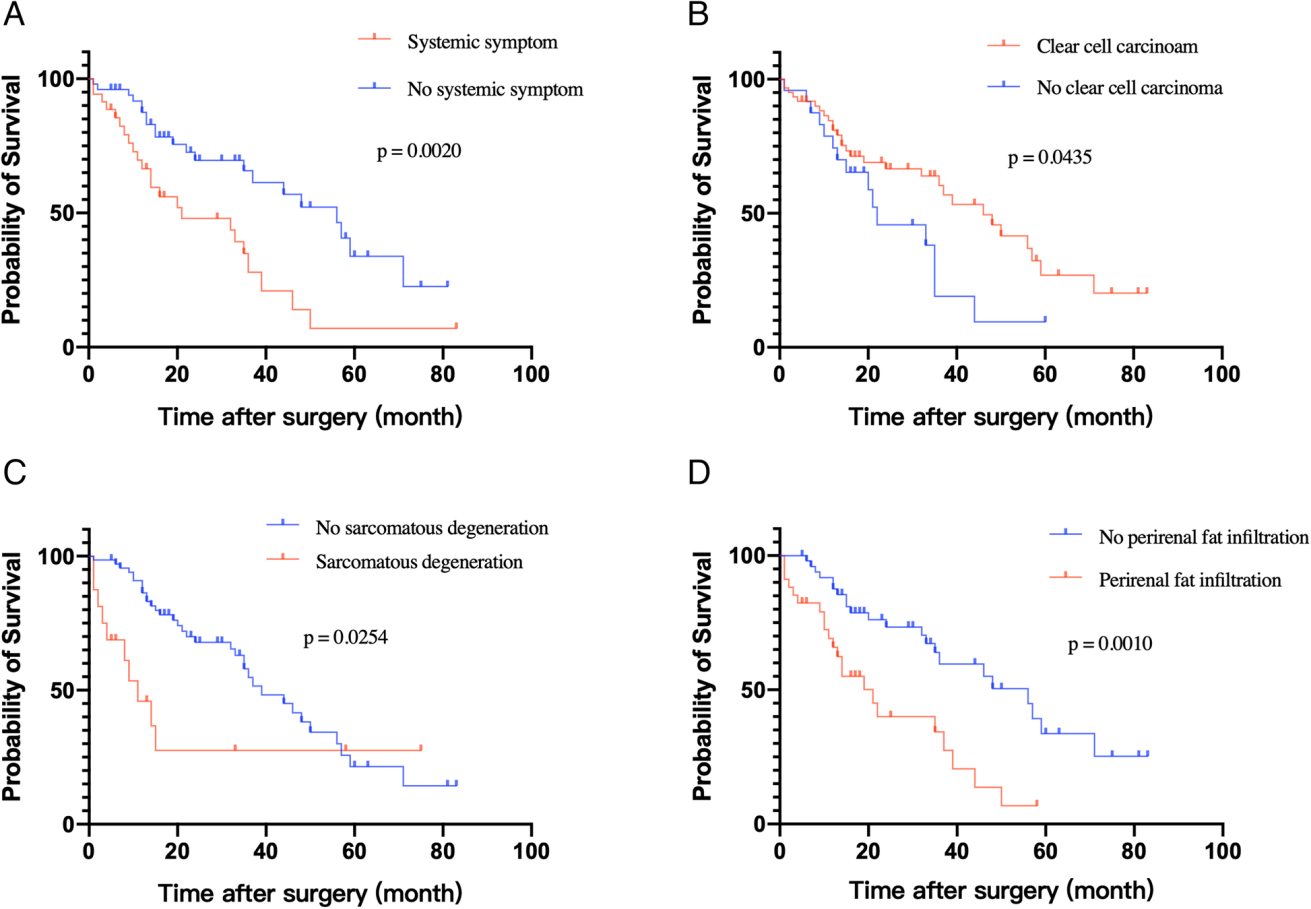
Kaplan–Meier survival curves in overall survival of patients with metastatic renal cell carcinoma with venous tumor thrombus who received cytoreductive nephrectomy **a** patients with systemic symptoms or not; **b** clear cell carcinoma or not; **c** sarcomatous degeneration of tumor or not: **d** perirenal fat infiltration or not

The results of the univariate and multivariate analyses evaluating the significance of different clinicopathological characteristics as predictors of OS in the 85 patients are shown in Table 2. A multivariable Cox proportional hazards model was used to investigate independent relationships with OS for variables that were available prior to surgery and were significant in univariate analyses. In multivariate analysis, systemic symptom (*p* = 0.00753), pathological type (*p* = 0.0166), sarcomatous degeneration (*p* = 0.0334), and perirenal fat infiltration (*p* = 0.0202) are independent predictors of OS. The OS of above subgroups was shown in Fig. 2. However, Mayo grade and multiple distant metastasis are not predictors of overall survival.

**Table 2:** Uni- and multivariate analyses of associations between various factors showing significance in log-rank test and overall survival in patients with metastatic renal cell carcinoma and tumor thrombus who received cytoreductive nephrectomy and thrombectomy

	Univariate analysis			Multivariate analysis		
Variables	HR	95%Cl	P	HR	95%Cl	P
Symptom (systemic symptom vs local symptom and no symptom)	2.52	1.38–4.63	0.00278	2.38	1.26–4.50	0.00753
Nuclear grading (1, 2, 3, vs 4)	1.52	0.668–3.45	0.319	0.989	0.401–2.44	0.980
Pathological type (clear cell carcinoma vs non-clear cell carcinoma)	1.62	1.08–2.43	0.0201	1.68	1.10–2.57	0.0166
Sarcomatous degeneration (yes vs no)	2.22	1.09–4.55	0.0287	2.30	1.07–4.94	0.0334
Renal hilar lymph node (yes vs no)	3.78	1.71–8.37	0.00102	1.55	0.603–3.97	0.364
Perirenal fat infiltration (yes vs no)	2.90	1.56–5.38	<0.001	2.33	1.14–4.74	0.0202

## Discussion

Although cytoreductive nephrectomy and thrombectomy is the standard treatment for patients with RCC and IVC TT [13, 16], there are still disputes about whether to perform surgical treatment or simply systemic therapy for these patients with distant metastasis. The invasion of IVC is one of the most significant variables determining the prognosis of patients with mRCC, according to prior research [6, 9, 17]. With the emergence of targeted drugs, the survival period of patients with mRCC is significantly longer than that of the era of cytokine therapy.

To date, there were few studies on the outcomes of targeted therapy sequential cytoreductive nephrectomy and tumor thrombectomy in mRCC patients with IVC TT. In previous studies, Westesson et al. indicated that Mayo grade had no significant effect on survival in patients with metastatic mRCC with TT [16]. In addition, Lenis et al. discovered that palliative resection was related to the improvement of OS rate in patients with TT confined to renal vein or inferior phrenic vena cava [9]. Entering targeted therapy, the choice of postoperative adjuvant therapy has a more significant impact on patient OS than TT level. However, considering the clinical symptoms caused by tumor thrombus, surgical treatment is also necessary [10]. Based on above findings, we retrospectively analyzed the OS in a total of 85 mRCC patients with TT who were treated with systemic therapy sequential cytoreductive nephrectomy and thrombectomy to verify the surgery safety and explore the adverse prognostic factors of these patients.

In present study, all patients received cytoreductive nephrectomy and thrombectomy and systemic treatment. The frequency of complications during the postoperative hospitalization was 32.9%, and the proportion of severe complications was 9.4%, including renal failure, urinary fistula, and pneumothorax, which seemed like a reasonable figure. The incidence of complications in our center is similar to previous literature [16]. It is worth noting that we recommend preoperative angiography evaluation of the left TT filled with the IVC to ensure the establishment of sufficient collateral circulation after surgery. Most patients with complications have achieved relief after receiving conservative treatment and have not died due to postoperative complications. Considering the impact of complications on the main outcomes of patients, the incidence of major complications in this study was 9.4%. For patients with grade 3 or above complications, more active intervention should be taken to prevent their impact on OS [9, 16].

Similar to the earlier findings, different Mayo grades have no impact on patients’ prognoses [8]. We believe that further sub stratification of T staging and TT levels is unlikely to increase further prognostic significance. Considering the effect of high Mayo grade TT on hemodynamics, surgery seems to be an alternative treatment, especially on the basis of the continuous development of cardiovascular bypass and veno-venous bypass technology [18], as well as the combination of robot-assisted surgery together with cardiovascular bypass [19]. In previous studies, some studies have shown an association between enlarged lymph nodes and poorer prognosis [20], but others have not [8]. Interestingly, hilar lymph node metastasis was shown to have an effect on survival in a univariate analysis but not in a multifactorial analysis, and we hypothesize that hilar lymph node enlargement is suggestive of a worse prognosis in specific patients, but is not an independent risk factor. When TT grows to the IVC, the patient is prone to local or systemic symptoms. Lumbago will affect the life and work of patients, and hematuria will lead to anemia and even hemorrhagic shock. Surgical treatment for such patients can significantly improve their quality of life. Interestingly, patients with clear cell carcinoma have a better prognosis after surgery. We speculate that this may be because patients are more sensitive to targeted drugs after surgery. The pathological results of sarcomatous degeneration and perirenal fat infiltration suggest a worse prognosis, which is consistent with previous literature [8]. The median OS of patients in this study was 33 months, slightly longer than in other studies, which may be due to a strict selection of patients suitable for surgery. The meta-analysis of Petrelli et al. showed that compared to patients receiving targeted therapy alone, mRCC patients receiving cytoreductive nephrectomy and targeted therapy had a reduced risk of death by more than 50% [21], which further emphasizes the significance of surgery. In the era of targeted therapy, we think surgical treatment is still necessary for patients with mRCC and TT due to the high incidence of clinical symptoms of these patients. Surgical treatment can alleviate clinical symptoms and improve the quality of life of patients to a certain extent.

This study still has some limitations. First of all, there was no comparison between surgical treatment and simple systemic treatment. Patients who chose simple systemic treatment were not included in the study due to lack of complete follow-up data. Secondly, this study is a retrospective study, and its conclusions need to be further verified by a prospective controlled study. Thirdly, different kinds of targeted drugs may have an impact on the prognosis after surgery. This study did not compare the prognosis between different targeted drugs. In the end, a few scholars believe that cytoreductive nephrectomy is of limited value in the treatment of mRCC [1, 6]. In the era of targeted therapy, the median survival time of tyrosine kinase inhibitors or mammalian target of rapamycin in the treatment of mRCC is 25–30 months [22]. The therapeutic value and necessity of cytoreductive nephrectomy is not widely recognized.

## Conclusion

In conclusion, we think that cytoreductive nephrectomy and thrombectomy is relatively safe and effective for patients with mRCC accompanied by IVC TT. In this series of patients, the worse prognosis is associated with systemic symptoms, non-clear cell carcinoma, sarcomatous degeneration, and perirenal fat infiltration.

## Data Availability

The datasets generated and/or analyzed during the current study are not publicly available due they are from clinical database at our medical center but are available from the corresponding author on reasonable request.

## Abbreviations

mRCC: Metastatic renal cell carcinoma
TT: Tumor thrombus
OS: Overall survival
IVC: Inferior vena cava
RCC: Renal cell carcinoma
CT: Computed tomography
MRI: Magnetic resonance imaging
PETCT: Positron emission tomography computed tomography
